# Limits to the appropriateness of intensive care: Policy statement of the German Interdisciplinary Association of Intensive Care and Emergency Medicine (DIVI)

**DOI:** 10.1007/s00063-018-0514-y

**Published:** 2018-11-30

**Authors:** G. Neitzke, H. Burchardi, G. Duttge, C. Hartog, R. Erchinger, P. Gretenkort, A. Michalsen, M. Mohr, F. Nauck, F. Salomon, H. Stopfkuchen, N. Weiler, U. Janssens

**Affiliations:** 1 Institute for Medical History, Ethics and Philosophy, Hannover Medical School, Hannover, Germany; 2 Bovenden, Germany; 3 Institute for Criminal Sciences—Department for Criminal Medical- and Biolaw, Georg-August-University Göttingen, Göttingen, Germany; 4 Department of Anesthesiology, Division of Operative Intensive Care Medicine, University Medicine Berlin, Berlin, Germany; 5 Schopp, Germany; 6 Simulation and Emergency Academy Helios Clinic Krefeld, Krefeld, Germany; 7 Department of Anaesthesiology, Intensive Care Medicine, Emergency Medicine and Pain Management, Tettnang Clinic Campus Bodensee, Tettnang, Germany; 8 Department of Anaesthesiology and Intensive Care Medicine, DIAKO Bremen, Bremen, Germany; 9 Department of Palliative Medicine, University Medicine Göttingen, Göttingen, Germany; 10 Lemgo, Germany; 11 Mainz, Germany; 12 Department of Anaesthesiology and Operative Intensive Care, University Schleswig-Holstein, Campus Kiel, Kiel, Germany; 13 Medical Clinic and Medical Intensive Care Medicine, St.-Antonius Hospital, Dechant-Decker-Str. 8, 52249 Eschweiler, Germany

## 1 Preamble

Intensive care medicine saves lives. New developments in medical technology and pharmacology have brought about a dimension of medical progress that now offers the chance of survival when formerly death was considered to be inevitable. However, the possibility and feasibility to sustain and prolong life is not devoid of controversy and tragic consequences, in particular when death cannot be averted despite the best efforts of ICU clinicians or when survival is associated with a substantial and permanent reduction of quality of life. Thus, in view of a poor prognosis, the question frequently arises whether continuation of intensive care therapy is still appropriate and meaningful or rather not. Where do we draw the line within the field of technically feasible measures? What are the perspectives and attitudes of the different parties involved in the treatment and affected by it?

The Ethics Section of the “Deutsche Interdisziplinäre Vereinigung für Intensiv- und Notfallmedizin, DIVI” (German Interdisciplinary Association for Intensive Care and Emergency Medicine) has prepared this policy statement as an aid to orientation for medical and nursing staff working in critical care medicine. With this paper, the Ethics Section wants to contribute to an informed and multi-professional discourse about the appropriateness of therapies and treatment limitations in intensive care units (ICU). The position paper shall help to examine expectations and hopes in individual cases by means of specific criteria. Thus, this paper illustrates the responsibility of the clinicians involved in the care of the respective patient.

The frequently employed Anglo-American term ‘*futility*’ will not be used here as it is inadequately defined and often limited to economic cost–benefit analyses. In the context of patient care, the question whether diagnostic or therapeutic measures are appropriate and meaningful should be settled without considering economic aspects. This position paper provides decision-making aids for such situations. Only treatment concepts that are considered to be appropriate qualify for implementation. After the appropriateness of therapy has been clarified in a particular case, the given legal provisions of cost effectiveness according to current German Social Act (Health Law) must also be observed. However, the process of determining the appropriateness of individual measures should not be mixed with the societal processes which define the larger health economic context. Economic considerations must not influence the process of determining appropriateness in a given case.

## 2 General considerations

### 2.1 Goals of intensive care medicine

In general, patients are admitted to the ICU for curative treatment. Intensive care medicine provides medical and nursing therapies, medical devices, expertise and high staffing ratios in order to gain time for recovery of impaired or failing vital organ functions. The main objective is to ensure that patients—even if they do not recover completely—can lead a life that is independent from ICU care. Thus, in case of success, intensive care medicine enables survival and the patient’s return to a life that is as independent and self-determined as possible [14]. However, time and again a patient becomes completely—and sometimes irreversibly—dependent on life-sustaining medical devices. In other cases, intensive care treatment results in survival with major mental and physical deficits that cause a considerable reduction in the patient’s quality of life and constitute a great and persistent burden for relatives [3, 8].

### 2.2 Appropriateness/inappropriateness

When discussing treatment approaches, the question whether or not a treatment is overall meaningful and appropriate must always be taken into account. This refers to the meaning and relevance of the therapeutic goal and the diagnostic, therapeutic or nursing measures derived from this goal. The questions about meaning and relevance cannot be answered in an objective way, but must take into consideration individual and subjective attitudes of the patients for instance about the meaning of life, death and suffering and assessments of quality of life, way of living and lifetime goals. These considerations take place both intuitively, based on “gut feeling” and by a reflective, rational process.

Activities or conditions are considered to be appropriate and meaningful if they are in some way relevant to achieve one’s (life) goals. What is regarded as a worthwhile life sustaining treatment by one person might be considered as a meaningless torture and mere prolongation of the dying process by another. Thus, the assessment of appropriateness in the sense of being meaningful permits different results for the same treatment measures. Depending on the point of view, the same measure may be rated as either “appropriate” or “inappropriate”. Between these two extremes, a continuum of assessments unfolds in the sense that something can be rated as more or less appropriate. The question about appropriateness includes two components: instrumental rationality and value rationality. Both can be considered and discussed separately.

Instrumental rationality describes the adequacy of a measure to attain a certain purpose/goal (example: “It is appropriate to treat this infection with antibiotics.”). In this sense, medical or nursing measures are appropriate/meaningful if sufficient experience or evidence exists to expect that the measure will bring about a success of treatment with a given probability.

By contrast, a measure is value rational if it expresses or asserts ethical values (example: “It is appropriate to help a patient who suffers from an infection.”). These fundamental values are culture- and time-dependent, and deeply rooted in the individual conceptions of mankind and moral attitudes. Therefore, several issues need to be clarified when assessing the appropriateness of a treatment, including questions about the value of the treatment goal, about the meaning of suffering and illness, about subjective assessments of quality of life, and about the significance of professional and family support.

Assessing the overall appropriateness of a measure always includes both components mentioned above. For this reason, the statement that a treatment option is appropriate/meaningful contains an assessment of its instrumental rationality (it is appropriate from a technical/scientific point of view) as well as of its value rationality (it is appropriate from a humane point of view). Attribution of appropriateness may vary depending on the perspective. Therefore, it must be assessed on a case-by-case basis how physicians, nursing staff, patients and relatives arrive at their evaluation of appropriateness/meaningfulness, and which consequences for treatment decisions arise from this assessment.

If the physician (after examination of the criteria listed in point 3.1) determines that a treatment option is appropriate, this option can be offered to the patient. Now it is up to the patient to assess the appropriateness of this option from his point of view (see 3.2). In case the physician does not recognize any degree of appropriateness for a certain therapeutic measure (after examination of the criteria in point 3.1), the treatment is not indicated and must not be offered. This medical judgment protects patients from inappropriate and pointless treatments.

## 3 Criteria of inappropriateness

When weighing the pros and cons of a measure, issues for consideration are not only objective facts but also intuitive and emotional valuations that are based on a number of different perceptions and attitudes. This applies to the professional point of view (see 3.1) as well as to the patient’s perspective (see 3.2), and these two sides may come to different conclusions regarding the appropriateness of a measure. A reflective understanding of criteria for appropriate treatment is a necessary requirement for qualified and sound decision making.

In many treatment situations it is difficult to justify or provide positive evidence for the appropriateness and meaningfulness of treatment concepts and measures. The inappropriateness of a certain treatment approach, however, is much easier to discern. For this reason, these recommendations list criteria for assessing potential inappropriateness. To assess whether a treatment concept or measure is inappropriate, the following questions need to be addressed:

- Can the intended goal of therapy be achieved from a medical point of view?
- Does the goal of therapy meet the patient’s wishes?
- Does the achievable quality of life justify the burdens that arise from the treatment from the patient’s point of view?

Treatment concepts or measures are inappropriate if

- the intended goal of therapy cannot be achieved,
- the intended goal of therapy is not supported by the patient’s wishes,
- from the patient’s view, the burden that arises from the treatment does not justify the achievable quality of life.

### 3.1 Professional perspective

#### 3.1.1 Goals of therapy

A goal of therapy is the intended result of a treatment. It is quite possible that different members of the team prefer different therapy goals. The following outcomes can be considered as goal of therapy:

- cure,
- prolongation of life,
- improving quality of life,
- symptom control,
- end of life care.

These different therapy goals cannot always be distinguished clearly.

Various factors influence the evaluation of therapy goals, including medical facts and reliable prognostic assessments, individual hopes and experiences, and also professional ambitions and medico-legal aspects. In case a therapy goal can de facto not be achieved according to best medical judgment, treatment measures that aim at this goal are inappropriate. If a therapy goal has a very low probability of success according to professional judgment, treatment measures intended to reach this goal are not a priori inappropriate but their indication should be seen as questionable and doubtful [9, 13]. In such situations, the physician must resolve the question of appropriateness from the patient’s point of view critically and in-depth with all persons involved.

In case a treatment measure is considered inappropriate, a change of therapy goal is unavoidable.

#### 3.1.2 Prognosis

A prognosis comprises the assessment of the likelihood of possible disease courses. This assessment is performed by the treating team and refers to clinical endpoints like survival, restoration of certain organ functions, quality of life or ability to communicate. A prognosis is based on relevant clinical findings, statistical probabilities and physician or nurse expertise; it does not represent a liable prediction of a patient’s individual course. Prognoses help to estimate whether therapy goals can be achieved and can thus serve as a measure for assessing their appropriateness.

Irreversibility of a medical condition is a major issue when providing a prognosis. Irreversibility is not so much a fact that can be ascertained in an objective way but rather a professional estimate that also includes subjective and individual assessments. Therefore, declaring irreversibility requires a broad consent within the multi-professional treating team. An exact prediction of a patient’s fate is impossible; therefore the tolerable remaining uncertainty needs to be determined in a consensus process (e. g. “With a probability approaching certainty, this condition is irreversible.”). By coming to a consensus about the irreversibility of a condition, the treating team takes over responsibility for the remaining minimal and acceptable probability of error.

Use of scoring systems for prognosis is only appropriate in the context of clinical studies where scores serve to describe patient cohorts, for quality improvement measures and for predictions based on large populations of intensive care patients. An individual prediction of a patient’s prognosis is not possible due to the remaining significant uncertainty.

#### 3.1.3 Patient wishes/autonomy

Interventions which are medically considered appropriate may only be carried out if they are in line with the patient’s stated or presumed wishes. For this reason, the patient’s wishes must be elicited by all means. On the other hand, decisions must not be driven by the treating team’s own values. If patients are mentally incapacitated, it is up to their legal proxy to find out and assert the patient’s preferences and wishes [9].

#### 3.1.4 Quality of life and burden

Present and future quality of life is intuitively assessed by the members of the treating team. However, this assessment must not serve as a basis for deciding whether further treatment is appropriate or not. This decision is reserved for the patient. In order to make sure that the patient can competently weigh his options, the information about possible disease courses and foreseeable impairment of the quality of life must be honest and comprehensible. Desired as well as undesirable treatment effects have to be discussed, including consequences which affect the patient as well as the relatives (e. g. survival without regaining consciousness).

### 3.2 Patient perspective

#### 3.2.1 Therapy goal

Assessing whether or not a therapy goal can be achieved is the task of the physician in charge. But it is up to the patient to decide if therapy goals which are achievable are also desirable and if they are to be pursued. This decision is influenced by the patient’s values, philosophical and religious beliefs, way of life and plans for the future, hopes and fears. In order to make a sound judgment about therapy goals that are in principle achievable, the patient or his legal representative have to be informed in detail by the physician about the medical condition which can be expected after the therapy goal is achieved.

#### 3.2.2 Prognosis

The patient or his legal proxy is informed about the prognosis. This means that they are informed about the individual probability of actually achieving those therapy goals (listed in 3.1.1) which are in principle achievable. In this context, it is necessary to point out the general uncertainty of prognosis. However, the evaluation of the prognosis is up to the patient. Consequently, the patient must decide whether or not a certain prognosis is acceptable from his point of view. The patient will agree to a further treatment only if he deems the prognosis acceptable and worthwhile.

From the patient’s perspective, the decision does not rest on the probability of success alone. It seems reasonable to consider a prognosis as good if the probability of success is high. In return, a low probability of success can justify a negative assessment. But even if the probability of success is low, many patients see reason for hope. From the patient’s point of view, whether hope exists or not does not depend on the factual probability alone, but also on the theoretical possibility of a positive outcome. The difference between optimism and hope should therefore be taken into account when deliberating with the patient or his legal representative.

#### 3.2.3 Patient wishes/autonomy

The patient’s right of self-determination is a valuable ethical achievement that is protected by law. The patient’s wishes have to be respected [5]. Concerning potentially inappropriate treatment this means: autonomy can only become effective if it is supported and empowered. This includes patient-oriented, open and unbiased patient information and the willingness to explain therapy goals and prognoses within a patient–doctor relationship based on mutual trust.

The patient’s wishes are not always unambiguous and may change in the course of treatment. Especially in case of competing or contradictory interests, repeated counseling and support from the treating team is necessary. There may be considerable ambivalence about the appropriateness of further treatment. In the process of consultation, the patient’s moral values and his presumed perspective on the progression of his disease should be addressed.

In case of inconsistencies in the patient’s wishes—e. g. between the patient’s advance directive and his consent to a recently performed operation, or if a patient who is not fully competent makes verbal statements that are discrepant to previously stated wishes—this inconsistency has to be clarified as far as possible: Do these current declarations of will constitute a revocation of the patient’s advance directive? Up to which extent do preoperative discussion and the patient’s consent to an operation justify subsequent life support and at which point in time is a new assessment and a new decision required that are in line with the previously stated patient wishes?

#### 3.2.4 Quality of life/burden

The WHO defines quality of life as “an individual’s perception of their position in life in the context of the culture and value systems in which they live, and in relation to their goals, expectations, standards and concerns” [15] (English definition available at www.who.int/mental_health/media/68.pdf). Accordingly, quality of life is an individual assessment that might change over time and that is difficult to ascertain in an objective way. However, the question: “How does the patient assess his current and his expected quality of life and does he accept the latter?” must not be ignored. This question cannot be answered but by the patient himself, and the answer only applies to the current situation. Assessment of the possible future quality of life is limited by the fact that this assessment can change when the future situation is actually experienced. For example, needing a wheelchair might be deemed unacceptable by a healthy person whereas the same person might come to an entirely different assessment when actually experiencing this situation.

Additional factors which play a role for the patient’s assessment are the physical and emotional burdens of intensive care therapy. For the patient, therapeutic measures can be painful and invasive and often involve a loss of control, privacy and personal rights. Acceptance of these impairments is higher when therapeutic measures are considered appropriate and meaningful. With declining degree of appropriateness, growing emphasis is placed on potential burdens and impairments when re-assessing therapy goals.

### 3.3 Treatment attempt

In case it is not possible to make a prognosis with sufficient certainty, a limited treatment attempt can help to come to a definite decision. In this case, a potentially appropriate treatment is started in order to see if it is successful. Even if the treatment is not successful or of doubtful success, the attempt helps all persons involved to come to a definite decision. Such a treatment attempt for the purpose of guiding future decision making must aim for a clearly defined therapy goal that is reasonably achievable. In addition, the therapy goal should be achievable within a limited period of time (e. g. “If cardiac function does not improve within one week, …”). Otherwise, there is the risk that a definite decision is delayed from one elusive hope to another.

## 4 Challenges of implementation

### 4.1 Decision-making under uncertainty

Medical prognoses provide an uncertain forecast for the individual course of disease. There always remains a certain degree of uncertainty the treating team needs to be aware of. Especially in intensive care medicine, a patient’s condition can change dramatically and unexpectedly. For this reason, prognoses as well as resulting decisions need to be scrutinized and readjusted regularly.

This uncertainty must not be ignored when talking to patients and relatives. It is necessary to explain all different possible courses the disease can take and to openly discuss resulting consequences. However, this is contrary to the need for unambiguousness of some patients and relatives. Medical professionals should avoid pretending such unambiguousness.

### 4.2 When successful treatment seems unlikely

In certain situations, the chances of a positive treatment outcome seem quite unlikely. The indication for life sustaining treatment cannot automatically be denied in these situations, but it remains doubtful and questionable.

First, therefore, the multi-professional treating team should reach a consensus. Subsequently, physician and ICU team must consider carefully together with the patient and/or his legal proxy by weighing up medically feasible treatment options against what is considered appropriate from the patient’s point of view, in order to define individual limitations of therapy [7, 9, 11].

This requires regular and open discussions between patient (and/or legal proxy), relatives and treating team [2]. Such a dialog permits all parties involved to come to a conclusion about the appropriateness of a specific therapy when the chances of success are very limited and the risk of medical complications or treatment burden is high.

When the probability of achieving a given therapy goal is unpredictable, but if there is no time for joint decision making due to high urgency, a treatment attempt appears justified. However, it is strongly recommended to re-evaluate the decision after a previously agreed interval.

### 4.3 When survival appears almost impossible

In case the patient’s survival appears impossible with a justifiable remaining uncertainty, the therapy goal must be changed towards palliation [12]. This decision should be disclosed to the patient and his relatives or to the legal proxy in an empathic and open manner in order to obtain their understanding and agreement if possible.

However, consent of the patient or his proxy is not legally required in such situations. If medical treatment is (or has become) inappropriate or impossible, there is—according to German jurisdiction—a priori no scope for the right of self-determination. A prerequisite for the dialog between the physician and the proxy is the presence of a given indication for therapy. The physician bears sole responsibility for the indication.

However, in individual cases, there might be reason for continuing inappropriate life sustaining treatments for a limited period of time. This includes the maintenance of organ function for planned organ donation [6] or bridging time so that relatives can bid farewell to the dying patient, as long as this does not contradict his or her will.

An unjustified wait-and-see attitude, i. e. avoiding or denying the decision to modify the therapy goal, is also a kind of decision. As this attitude can cause the patient considerably more suffering than benefit in the final phase of life, it is not acceptable [9].

### 4.4 When the therapy goal is inappropriate from the patient’s point of view

Sometimes the patient does not agree to therapy goals that are achievable from a medical point of view because they are not in line with his idea of a life worth living. It might also be possible that a patient rejects a therapy goal because he does not accept the treatment burden. In both cases, treatment is inappropriate and meaningless from the patient’s point of view and must not be provided.

The treating team has to realize that regardless of medical results and criteria, the patient might question the appropriateness of medically indicated therapies because of his individual assessments. Amongst others, these include ideas of a successful life, dying with dignity, significance of death, individual life goals and aspects of quality of life. In any case, before life sustaining treatment is withdrawn on request of the patient, it has to be ensured that the patient’s assessment of the situation is not based on a temporary mood or caused by his illness. In case of doubt, an independent professional assessment (for instance by a psychiatrist) should be carried out.

If the patient considers that further treatment is meaningless and inappropriate, ignoring the patient’s will cannot be justified by the claim that the physician is fulfilling a presumed obligation to help, at least according to German legal stipulations. Rather, further treatment would constitute an offence of criminal assault.

### 4.5 Procedure in cases of conflict

Differences in opinion may arise among the persons involved about whether therapy goals are achievable or whether treatment options are appropriate; these differences can delay or even foil necessary decision making. Within the treating team, varying professional judgment can lead to conflicts on different levels of hierarchy as well as among medical specialties and health care professions. Health care providers should always check in a self-critical way if irrelevant motives or competing interests might influence their assessment [16]. Conflicts can also arise among relatives or between relatives and the treating team.

Personal involvement, emotional distress and the perception of being at the mercy of the treating team might make it difficult for patients and relatives to focus on relevant issues and to formulate and ask relevant questions. However, the appropriateness of therapy goals and measures can only be adequately assessed if all stakeholders are involved. It is the treating team’s task to provide an adequate atmosphere for this assessment, regardless of their heavy workload. Likewise, each member of the treating team—whatever their professional group or level of hierarchy—must be allowed to ask questions about the appropriateness of intensive care measures and to voice their concerns about possible influence of external forces and motives on therapy decisions.

Over all, good interdisciplinary and interprofessional communication is critical and should also be practiced by the team leaders in order to avoid and resolve conflicts. Good communication includes regular team meetings, if necessary together with colleagues from other departments, and structured discussions with relatives [7]. External assistance can be requested in form of an ethics case consultation by clinical ethics committees [4], or palliative care consultation [10].

Uncertainty about the implications of legal regulations concerning end-of-life decisions [1] can have the effect that a written or orally expressed patient wish, and thus the patient’s autonomous will, is disregarded. Regular pre- and postgraduate education on ethical, legal and palliative care issues, accompanied by continuous self-reflection, can help to provide clarity.

In cases of conflict within the family about meaningfulness and appropriateness of life-sustaining measures, the ICU team is usually not—or only to a very limited extent—able to help resolve these conflicts. However, even if a consensus cannot be reached, the assessment of appropriateness in terms of instrumental rationality remains the task and responsibility of physicians.

## 5 Decisional support

Evaluating the appropriateness and meaningfulness of therapy goals and treatment measures requires an assessment by all persons concerned. Whether the probability of success is *sufficiently* high, or whether the patient’s expressed wishes have been *sufficiently* ascertained, has to be resolved individually and cannot always be confirmed objectively. The following questions can be used as an aid to contribute towards these assessments.

### 5.1 Questions to be considered among the treating team

- Do you think it is likely that the patient will survive the current situation?
- Is the prognosis of survival based on objective assessment methods?
- Do you believe that in case of survival, the patient will regain a health condition that meets his ideas of an acceptable quality of life?
- Does an advance directive exist that clarifies the patient’s position?
- Do you have any other information about the patient’s expressed or presumed wishes?
- Do you assume that the current measures of therapy are appropriate from the point of view of the patient to improve his health condition and his quality of life?
- Do you think it is possible that the current diagnostic and therapeutic measures are an unacceptable burden for the patient and will further deteriorate the patient’s quality of life?
- Do you expect an improvement of the current situation (in terms of improvement of the quality of life during the current therapy)?
- Which short-term therapy goals would indicate an improvement?
- Do you perceive any circumstances (hopes and expectations within the team, of individual team members, of relatives) that make it difficult to assess the appropriateness or inappropriateness of therapy?
- Have such circumstances already been discussed between all persons concerned?
- Do all persons concerned share the treating physician’s assessment of appropriateness or inappropriateness?

### 5.2 Questions supporting self-reflection of proxies or relatives

- What are your own wishes? What are your wishes for the patient? What do you presume the patient wishes himself?
- Are you aware of the fact that it is highly unlikely that the patient survives this stage of disease?
- Are you aware of the reduction in quality of life in case the patient survives this stage of disease (for instance dependency on care, inability to communicate)?
- Of what significance are these considerations for your assessment?
- Did you check if the current medical situation and the current therapy goal are in line with the patient’s advance directive or with the preferences the patient expressed in the past?
- What other statements of the patient in the past (apart from the patient’s advance directive) could be relevant to find out what the patient might want in the current situation?
  Examples: How did he handle diseases? What were his experiences with death and dying of others? What was important to him in life? What was his conception of quality of life? What life plans did he have at last?
- Do you feel sufficiently informed for assessing the appropriateness and meaningfulness from the patient’s point of view?

It is strongly advised to document the results from these reflections in a structured way so that all relevant considerations and assessments are available for all persons concerned.
